# Community Analysis of a Crisis Response Network

**DOI:** 10.1177/0894439319858679

**Published:** 2019-07-28

**Authors:** Yushim Kim, Jihong Kim, Seong Soo Oh, Sang-Wook Kim, Minyoung Ku, Jaehyuk Cha

**Affiliations:** 1 Arizona State University, Phoenix, AZ, USA; 2 Hanyang University, Seoul, South Korea; 3 John Jay College, New York, NY, USA

**Keywords:** epidemic response network, community detection algorithms, network analysis, emergency management

## Abstract

This article distinguishes between clique family subgroups and communities in a crisis response network. Then, we examine the way organizations interacted to achieve a common goal by employing community analysis of an epidemic response network in Korea in 2015. The results indicate that the network split into two groups: core response communities in one group and supportive functional communities in the other. The core response communities include organizations across government jurisdictions, sectors, and geographic locations. Other communities are confined geographically, homogenous functionally, or both. We also find that whenever intergovernmental relations were present in communities, the member connectivity was low, even if intersectoral relations appeared together within them.

Many countries have struggled with coordinating efforts among diverse organizations in response to crises attributable to disasters and emergencies (Boin & ‘T Hart, 2010; Christensen, Ole, LÆgreid, & Rykkja, 2016). Disaster and emergency response network (ERN) studies approach the problem by identifying the interrelations (relation interdependencies) among organizations in a volatile environment—that is, the outcomes of emergency response as “a product of the attributes of the network” (Hossain & Kuti, 2010, p. 764). For example, some ERN studies identify cohesive subgroups in the Hurricane Katrina case using the definition of a clique (Comfort & Haase, 2006; Kapucu, 2005). However, the literature has not clearly articulated the basic theoretical and analytical rationales of finding cohesive subgroups in ERNs using cliques.

In this article, we distinguish between clique family subgroups and communities in ERNs. Conceptually, a clique is “…a group [in which] all members of which are in contact with each other or are friends, know each other, etc.” which generally refers to a social circle (Mokken, 1979, p. 161), while a community is formed through concrete social relationships (e.g., high school friends) or sets of people perceived to be similar, such as the Italian community and Twitter community (Gruzd, Wellman, & Takhteyev, 2011; Hagen, Keller, Neely, DePaula, & Robert-Cooperman, 2018). In social network analysis, a clique is operationalized as “…a subset of actors in which every actor is adjacent to every other actor in the subset (Borgatti, Everett, & Johnson, 2013, p. 183), while communities refer to “…groups within which the network connections are dense, but between which they are sparser” (Newman & Girvan, 2004, p. 69). The clique and its variant definitions (e.g., *n-cliques* and *k-cores*) focus on internal edges, while the community is a concept based on the distinction between internal edges and the outside. We argue that community analysis can provide useful insights about the interrelations among diverse organizations in the ERN.

We have not yet found any studies that have investigated cohesive subgroups in large multilevel, multisectoral ERNs through a community lens. With limited guidance from the literature on ERNs, we lack specific expectations or hypotheses about what the community structure in the network may look like. Therefore, our study focuses on identifying and analyzing communities in the 2015 Middle East Respiratory Syndrome Coronavirus (MERS) response in South Korea as a case study. We address the following research questions: (1) In what way were distinctive communities divided in the ERN? and (2) How did the interorganizational relations relate to the internal characteristics of the communities? By detecting and analyzing the community structure in an ERN, we offer insights for future empirical studies on ERNs.

## Cohesive Subgroups in ERNs

The interrelations in ERNs have been examined occasionally by analyzing the entire network’s structure. For example, the Katrina case exhibited a large and sparse network,^1^ in which a small number of nodes had a large number of edges and a large number of nodes had a small number of edges (Butts, Acton, & Marcum, 2012). The Katrina response network can be thought of as “…a loosely connected set of highly cohesive clusters, surrounded by an extensive ‘halo’ of pendant trees, small independent components, and isolates” (Butts et al., 2012, p. 23). The network was sparse and showed a tree-like structure but also included cohesive substructures. Other studies on the Katrina response network have largely concurred with these observations (Comfort & Haase, 2006; Kapucu, Arslan, & Collins, 2010).

In identifying cohesive subgroups in the Katrina response network, these studies rely on the analysis of *cliques*: “a maximal complete subgraph of three or more nodes” (Wasserman & Faust, 1994, p. 254) or clique-like (*n-cliques* or *k-cores*). The *n-cliques* can include nodes that are not in the clique but are accessible. Similarly, *k-cores* refer to maximal subgraphs with a minimum degree of at least *k*. Many cliques were identified in the Katrina response network, in which federal and state agencies appeared frequently (Comfort & Haase, 2006; Kapucu, 2005). Using *k-cores* analysis, Butts, Acton, and Marcum (2012) suggest that the Katrina response network’s inner structure was built around a small set of cohesive subgroups that was divided along institutional lines corresponding to five state clusters (Alabama, Colorado, Florida, Georgia, and Virginia), a cluster of U.S. federal organizations, and one of nongovernmental organizations. While these studies suggest the presence of cohesive subgroups in ERNs, we have not found any research that thoroughly discussed subsets of organizations’ significance in ERNs. From the limited literature, we identify two different, albeit related, reasons that cohesive subgroups have interested ERN researchers.

### Why Do Cohesive Subgroups in ERNs Matter?

In their analysis of cohesive subgroups using cliques, Comfort and Haase (2006) assume that a cohesive subgroup can facilitate achieving shared tasks *as a group*, but it can be less adept at managing the full flow of information and resources *across groups* and thus decreasing the entire network’s coherence. Kapucu and colleagues (2010) indicate that the recurrent patterns of interaction among the sets of selected organizations may be the result of excluding other organizations in decision-making, which may be a deterrent to all organizations’ harmonious concerted efforts in disaster responses. Comfort and Haase (2006) view cliques as an indicator of “…the difficulty of enabling collective action across the network” (p. 339),^2^ and others have adhered closely to this perspective (Celik & Corbacioglu, 2016; Hossain & Kuti, 2010; Kapucu, 2005). Cohesive subgroups such as cliques *are assumed to* be a potential hindrance to the entire network’s performance.

The problem with this perspective is that one set of eyes can perceive cohesive subgroups in ERNs as a barrier, while another can regard them as a facilitator of an effective response. While disaster and emergency response plans are inherently limited and not implemented in practice as intended (Clarke, 1999), stakeholder organizations’ responses may be performed together with presumed structures, particularly in a setting in which government entities are predominant. For example, the Incident Command System (ICS)^3^ was designed to improve response work’s efficiency by constructing a standard operating procedure (Moynihan, 2009). Structurally, one person serves as the incident commander who is responsible for directing all other responders (Kapucu & Garayev, 2016). ICS is a somewhat hierarchical command-and-control system with functional arrangements in five key resources and capabilities—that is, command, operations, planning, logistics, and finance (Kapucu & Garayev, 2016). In an environment in which such an emergency response model is implemented, it is realistic to expect clusters and subgroups to reflect the model’s structural designs and arrangements, and they may be intentionally designed to facilitate coordination, communication, and collaboration with other parts or subgroups efficiently in a large response network.

Others are interested in identifying cohesive subgroups because they may indicate a lack of cross-jurisdictional and cross-sectoral collaboration in ERNs. During these responses, public organizations in different jurisdictions participate, and a sizable number of organizations from nongovernmental sectors also become involved (Celik & Corbacioglu, 2016; Comfort & Haase, 2006; Kapucu et al., 2010; Spiro, Acton, & Butts, 2013). Organizational participation by multiple government levels and sectors is often necessary because knowledge, expertise, and resources are distributed in society. Participating organizations must collaborate and coordinate their efforts. However, studies have suggested that interactions in ERNs are limited and primarily occur among similar organizations, particularly within the same jurisdiction. That is, public organizations tend to interact more frequently with other public organizations in specific geographic locations (Butts et al., 2012; Hossain & Kuti, 2010; Kapucu, 2005; Tang, Deng, Shao, & Shen, 2017). These studies indicate that organizations have been insufficiently integrated across government jurisdictions (Tang et al., 2017) or sectors (Butts et al., 2012; Hossain & Kuti, 2010), and the identification of cliques composed of similar organizations reinforces such a concern.

In our view, there is a greater, or perhaps more interesting, question related to the cross-jurisdictional and cross-sectoral integration in interorganizational response networks: How are intergovernmental relations mixed with intersectoral relations in ERNs? Here, we use the term interorganizational relations to refer to both intergovernmental and intersectoral relations. Intergovernmental relations refer to the interaction among organizations across different government levels (local, provincial, and national), and intersectoral relations involve the interaction among organizations across different sectors (public, private, nonprofit, and civic sectors). Recent studies have suggested that both intergovernmental and intersectoral relations shape ERNs (Kapucu et al., 2010; Kapucu & Garayev, 2011; Tang et al., 2017), but few have analyzed the way the two interorganizational relations intertwine. If the relation interdependencies in the entire network are of interest to ERN researchers, as is the case in this article, focusing on cliques may not necessarily be the best approach to the question because clique analysis may continue to find sets of selected organizations that are tightly linked for various reasons.

### How to Find Cohesive Subgroups in ERNs

The analysis of cliques is a very strict way of operationalizing cohesive subgroups from a social network perspective (Moody & Coleman, 2015), and there are two issues with using it to identify cohesive subgroups in ERNs. First, clique analysis assumes complete connections of three or more subgroup members, while real-world networks tend to have many small overlapping cliques that do not represent distinct groups (Moody & Coleman, 2015). Even if substantively meaningful cliques appear, they may not necessarily imply a lack of information flow across subgroups or other organizations’ exclusion, as previous ERN studies have assumed (Comfort & Haase, 2006; Kapucu et al., 2010). Second, clique analysis assumes no internal differentiation in members’ structural position within the subgroup (Wasserman & Faust, 1994). In a task-oriented network such as an ERN, organizations within a subgroup may look similar (e.g., all fire organizations). However, this does not imply that they are identical in their structural positions. When these assumptions in clique analysis do not hold, identifying cohesive subgroups as cliques is inappropriate (Wasserman & Faust, 1994). Similarly, other clique-like approaches (*n-cliques* and *k*-*cores*) demand an answer to the question: “What is the *n-* or *k-*?” The clique and clique-like approaches have a limited ability to define and identify cohesive subgroups in a task-oriented network because they do not clearly explain why the subgroups need to be defined and identified in such a manner. We proposed a different way of thinking about and finding subsets of organizations in ERNs: community.

When a network consists of subsets of nodes with many edges that connect nodes of the same subset, but few that lay between subsets, the network is said to have a community structure (Wilkinson & Huberman, 2004). Network researchers have developed methods with which to detect communities (Fortunato, Latora, & Marchiori, 2004; Latora & Marchiori, 2001; Lim, Kim, & Lee, 2016; Newman & Girvan, 2004; Yang & Leskovec, 2014). Optimization approaches, such as the Louvain and Leiden methods, which we use in this article, sort nodes into communities by maximizing a clustering objective function (e.g., modularity). Beginning with each node in its own group, the algorithm joins groups together in pairs, choosing the pairs that maximize the increase in modularity (Moody & Coleman, 2015). This method performs an iterative process of node assignments until modularity is maximized and leads to a hierarchical nesting of nodes (Blondel, Guillaume, Lambiotte, & Lefebvre, 2008). Recently, the Louvain algorithm was upgraded and improved as the Leiden algorithm that addresses some issues in the Louvain algorithm (Traag, Waltman, & van Eck, 2018).

Modularity (*Q*), which shows the quality of partitions, is measured and assessed quantitatively:

1 $$Q=\overset{k}{\underset{i}{\sum }}\left(e_{ii}-{\left(\overset{k}{\underset{j}{\sum }}e_{ij}\right)}^{2}\right),$$

in which $e_{ii}$ is the fraction of the intra-edges of community *i* over all edges, and $e_{ij}$ is the fraction of the inter-edges between community *i* and community *j* over all edges. Modularity scores are used to compare assignments of nodes into different communities and also the final partitions. It is calculated as a normalized index value: If there is only one group in a network, *Q* takes the value of zero; if all ties are within separate groups, *Q* takes the maximum value of one. Thus, a higher *Q* indicates a greater portion of intra- than inter-edges, implying a network with a strong community structure (Fortunato et al., 2004).

Currently, there are two challenges in community detection studies. First, the modular structure in complex networks usually is not known *beforehand* (Traag et al., 2018). We know the community structure only after it is identified. Second, there is no formal definition of community in a graph (Reichardt & Bornholdt, 2006; Wilkinson & Huberman, 2004), it simply is a concept of relative density (Moody & Coleman, 2015). A high modularity score ensures only that “…the groups as observed are distinct, not that they are internally cohesive” (Moody & Coleman, 2015, p. 909) and does not guarantee any formal limit on the subgroup’s internal structure. Thus, internal structure must be examined, especially in such situations as ERNs.

Despite these limitations, efforts to reveal underlying community structures have been undertaken with a wide range of systems, including online and off-line social systems, such as an e-mail corpus of a million messages in organizations (Tyler, Wilkinson, & Huberman, 2005), Zika virus conversation communities on Twitter (Hagen et al., 2018), and jazz musician networks (Gleiser & Danon, 2003). Further, one can exploit complex networks by identifying their community structure. For example, Salathé and Jones (2010) showed that community structures in human contact networks significantly influence infectious disease dynamics. Their findings suggest that, in a network with a community structure, targeting individuals who bridge communities for immunization is better than intervening with highly connected individuals.

### Difficulty in Characterizing Communities in ERNs

We exploit the community detection and analysis to understand an ERN’s substructure in the context of an infectious disease outbreak. It is difficult to know the way communities in ERNs will form beforehand without examining clusters and their compositions and connectivity in the network. We may expect to observe communities that consist of diverse organizations because organizations’ shared goal in ERNs is to respond to a crisis by performing necessary tasks (e.g., providing mortuary and medical services as well as delivering materials) through concerted efforts on the part of those with different capabilities (Moynihan, 2009; Waugh, 2003). Organizations that have different information, skills, and resources may frequently interact in a disruptive situation because one type alone, such as the government or organizations in an affected area, cannot cope effectively with the event (Waugh, 2003). On the other hand, we also cannot rule out the possibility shown in previous studies (Butts et al., 2012; Comfort & Haase, 2006; Kapucu, 2005). Organizations that work closely in normal situations because of their task similarity, geographic locations, or jurisdictions may interact more frequently and easily, even in disruptive situations (Hossain & Kuti, 2010), and communities may be identified that correspond to those factors. A case could be made that communities in ERNs consist of heterogeneous organizations, but a case could also be made that communities are made up of homogeneous organizations with certain characteristics.

It is equally difficult to set expectations about communities’ internal structure in ERNs. We can expect that, regardless of their types, sectors, and locations, some organizations work and interact closely—perhaps even more so in such a disruptive situation. Emergent needs for coordination, communication, and collaboration also can trigger organizational interactions that extend beyond the usual or planned structure. Thus, the relations among organizations become dense and evolve into the community in which every member is connected. On the other hand, a community in the task network may not require all of the organizations within it to interact. For example, if a presumed structure is strongly established, organizations are more likely to interact with others within the planned structure following the chain of command and control. Even without such a structure, government organizations may coordinate their responses following the existing chain of command and control in their routine. We may expect to observe communities with a sparse connection among organizations. Thus, the way communities emerge in ERNs is an open empirical question that can be answered by examining the entire network.

## Case Study

### 2015 MERS Response, South Korea

Several countries have experienced novel infectious disease outbreaks over the past decade (Silk, 2018; Swaan et al., 2018; Williams et al., 2015) and efforts to control such events have been more or less successful, depending upon the instances and countries. In low probability, high-consequence infectious diseases such as the 2015 MERS outbreak in South Korea, a concerted response among individuals and organizations is virtually the only way to respond because countermeasures—such as vaccines—are not readily available. Thus, to achieve an effective response, it is imperative to understand the way individuals and organizations mobilize and respond in public health emergencies. However, the response system for a national or global epidemic is highly complex (Hodge, 2015; Sell et al., 2018; Williams et al., 2015) because of several factors: (1) the large number of organizations across multiple government levels and sectors, (2) the diversity of and interactions among organizations for the necessary (e.g., laboratory testing) or emergent (e.g., hospital closure) tasks, and (3) concurrent outbreaks or treatments at multiple locations attributable to the virus’s rapid spread. All of these factors create challenges when responding to public health emergencies.

### Data Sources and Collection

We broadly define a response network as the relations among organizations that can act as critical channels for information, resources, and support. When two organizations engage in any MERS-specific response interactions, they are considered to be related in the response. Examples of interactions include taking joint actions, communicating with each other, or sharing crucial information and resources (i.e., exchanging patient information, workforce, equipment, or financial support) related to performing the MERS tasks, as well as having meetings among organizations to establish a collaborative network.

We collected response network data from the following two archival sources: (1) news articles from South Korea’s four major newspapers^4^ published between May 20, 2015, and December 31, 2015 (the outbreak period), and (2) a postevent white paper that the Ministry of Health and Welfare published in December 2016. In August 2016, Hanyang university's research center in South Korea provided an online tagging tool for every news article in the country’s news articles database that included the term “MERS (http://naver.com).” A group of researchers at the Korea Institute for Health and Social Affairs wrote the white paper (488 pages, plus appendices) based on their comprehensive research using multiple data sources and collection methods. The authors of this article and graduate research assistants, all of whom are fluent in Korean, were involved in the data collection process from August 2016 to September 2017.

Because of the literature’s lack of specific guidance on the data to collect from archival materials to construct interorganizational network data, we collected the data through trial and error. We collected data from news articles through two separate trials (a total of 6,187 articles from the four newspapers). The authors and a graduate assistant then ran a test trial between August 2016 and April 2017. In July 2017, the authors developed a data collection protocol based on the test trial experience collecting the data from the news articles and white paper. Then, we recollected the data from the news articles between August 2017 and September 2017 using the protocol.^5^

When we collected data by reviewing archival sources, we first tagged all apparent references within the source text to organizations’ relational activities. Organizations are defined as “any named entity that represents (directly or indirectly) multiple persons or other entities, and that acts as a *de facto* decision making unit within the context of the response” (Butts et al., 2012, p. 6). If we found an individual’s name on behalf of the individual’s organization (e.g., the secretary of the Ministry of Health and Welfare), we coded the individual as the organization’s representative. These organizational interactions were coded for a direct relation based on “whom” to “whom” and for “what purpose.” Then, these relational activity tags were rechecked. All explicit mentions of relations among organizations referred to in the tagged text were extracted into a sociomatrix of organizations.

We also categorized individual organizations into different “groups” using the following criteria. First, we distinguished the entities in South Korea from those outside the country (e.g., World Health Organization [WHO], Centers for Disease Control and prevention [CDC]). Second, we sorted governmental entities by jurisdiction (e.g., local, provincial/metropolitan, or national) and then also by the functions that each organization performs (e.g., health care, police, fire). For example, we categorized local fire stations differently from provincial fire headquarters because these organizations’ scope and role differ within the governmental structure. We categorized nongovernmental entities in the private, nonprofit, or civil society sectors that provide primary services in different service areas (e.g., hospitals, medical waste treatment companies, professional associations). At the end of the data collection process, 69 organizational groups from 1,395 organizations were identified (see Appendix).^6^

### Community Detection and Analysis

We employed the Leiden algorithm using Python (Traag et al., 2018), which we discussed in the previous section. The Leiden algorithm is also available for Gephi as a plugin (https://gephi.org/). After identifying communities, the network can be reduced to these communities. In generating the reduced graph, each community appears within a circle, the size of which varies according to the number of organizations in the community. The links between communities indicate the connections among community members. The thickness of the lines varies in proportion to the number of pairs of connected organizations. This process improves the ability to understand the network structure drastically and provides an opportunity to analyze the individual communities’ internal characteristics such as the organizations’ diversity and their connectivity for each community.

Shannon’s Diversity Index (*H*) is used as a measure of diversity because uncertainty increases as species’ diversity in a community increases (DeJong, 1975). The *H* index accounts for both species’ richness and evenness in a community (organizational groups in a community in our case). *S* indicates the total number of species. The fraction of the population that constitutes a species, *i*, is represented by *p_i_* below and then multiplied by the natural logarithm of the proportion ($\text{ln}p_{i}$). The resulting product is then summed across species and multiplied by −1:

2 $$H=-\overset{S}{\underset{i=1}{\sum }}p_{i}\times \text{ln}\;p_{i}.$$

High *H* values represent more diverse communities. Shannon’s *E* is calculated by  E=H/lnS, which indicates various species’ equality in a community. When all of the species are equally abundant, maximum evenness (i.e., 1) is obtained.

While limited, density and the average clustering coefficient can capture the basic idea of a subgraph’s structural cohesion or “cliquishness” (Moody & Coleman, 2015). A graph’s density (*D*) is the proportion of possible edges presented in the graph, which is the ratio between the number of edges present and the maximum possible. It ranges from 0 (no edges) to 1 (if all possible lines are present). A graph’s clustering coefficient (*C*) is the probability that two neighbors of a node are neighbors themselves. It essentially measures the way a node’s neighbors form a *1-clique*. *C* is 1 in a graph connected fully.

## Results

### MERS Response Network

The MERS response network in the data set consists of 1,395 organizations and 4,801 edges. Table 1 shows that most of the organizations were government organizations (approximately 80%) and 20% were nongovernmental organizations from different sectors. Local government organizations constituted the largest proportion of organizations (68%). Further, one international organization (i.e., WHO) and foreign government agencies or foreign medical centers (i.e., CDC, Erasmus University Medical Center) appeared in the response network.

**Table 1.** Profiles of Organizations.

	#	%
Government organizations (1,102)		
National level	55	3.9
Provincial/metropolitan level	90	6.5
Local level	954	68.4
Assembly (national, provincial, local)	3	0.2
Nongovernment organizations (271)		
Hospitals	182	13.0
Private companies	42	3.0
Political parties	2	0.1
Nongovernmental organizations	3	0.2
Civil society organizations	2	0.1
Academic/professional associations	29	2.1
Education institutions	11	0.8
International or foreign organizations (22)		
International organization	1	0.1
Foreign organizations	21	1.5
	1,395	100.0

Organizations coordinated with approximately three other organizations (average degree: 3.44). However, six organizations coordinated with more than 100 others. The country’s health authorities, such as the Ministry of Health and Welfare (MOHW: 595 edges), Central MERS Management Headquarters (CMMH: 551 edges), and Korea Centers for Disease Control and Prevention (KCDC: 253 edges), were found to have a large number of edges. The Ministry of Environment (304 edges) also coordinated with many other organizations in the response. The National Medical Center had 160 edges, and the Seoul Metropolitan City Government had 129.

### In What Way Were Distinctive Communities Divided?

**Figure 1.**
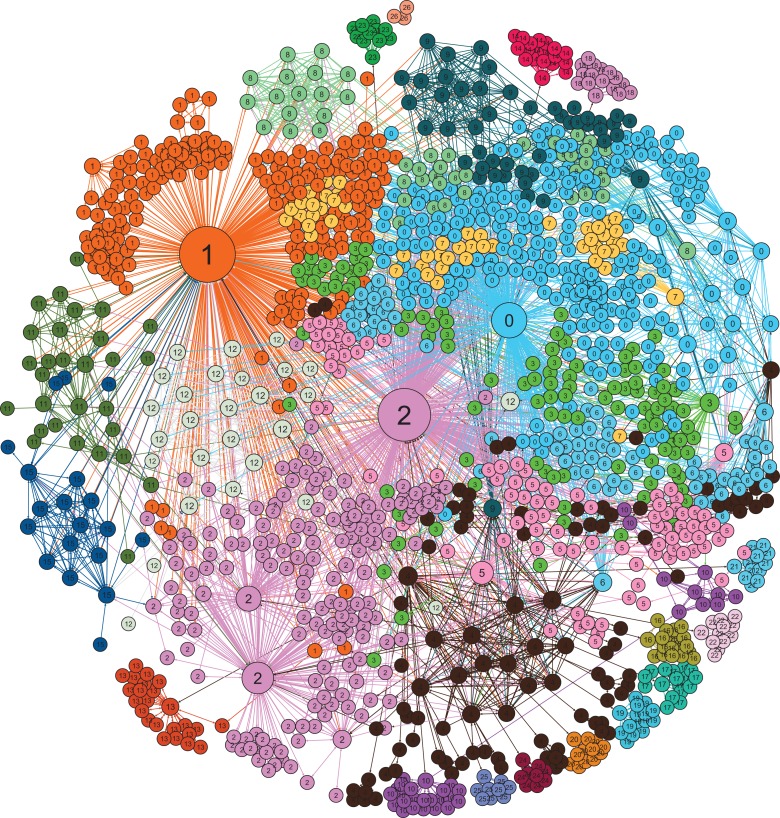
Communities in the Middle East Respiratory Syndrome Coronavirus response network. Communities were identified using the Leiden algorithm: quality function = modularity, resolution = 1.0, number of iterations = 1,000, number of restarts = 1, random seed = 0. Modularity score (*Q*) = 0.584.

**Figure 2. fig2-0894439319858679:**
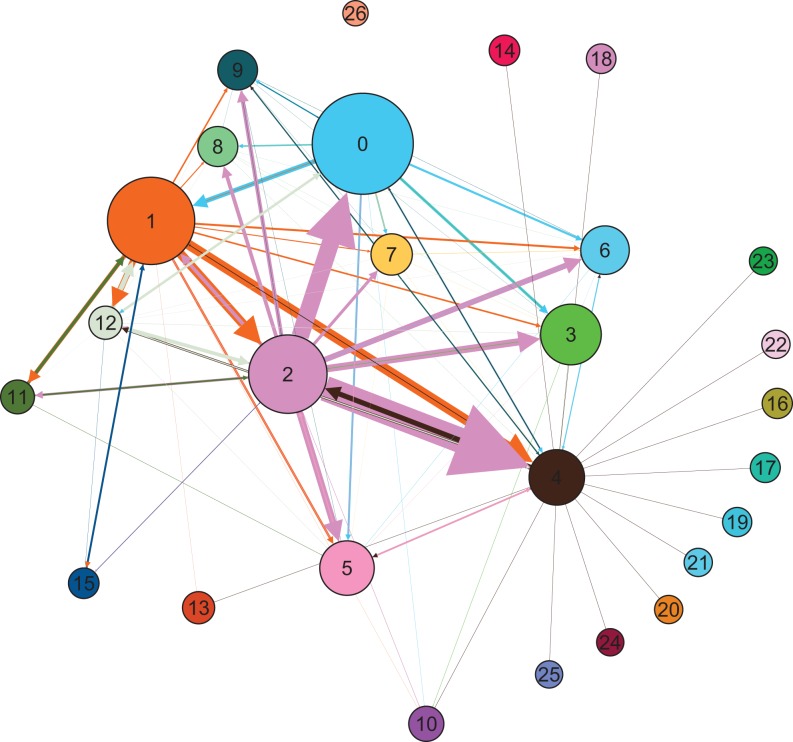
Reduced graph. Each community appears within a circle, the size of which varies according to the number of organizations in the community. The links between communities indicate the connections among community members. The thickness of the lines varies in proportion to the number of pairs of connected organizations.

The Leiden algorithm detected 27 communities in the network, labeled as 0 through 26 in Figures 1 [Fig fig2-0894439319858679]–3 and Tables 2 and 3. The final modularity score (*Q*) was 0.584, showing that the community detection algorithm partitioned and identified the communities in the network reasonably well. In real-world networks, modularity scores “…typically fall in the range from about 0.30 to 0.70. High values are rare” (Newman & Girvan, 2004, p. 7). The number of communities was also consistent in the Leiden and Louvain algorithms (26 communities in the Louvain algorithm). The modularity score was slightly higher in the Leiden algorithm than the *Q* = 0.577 in the Louvain.

**Table 2.** Community Analysis.

	Organization	Organization Groups	Member Diversity		Member Connection			
ID	#	#	*H*	*E*	*D*	*C*	Descriptions	Note
0	234	7	1.18	.60	.01	.01	Mixed organizations led by Ministry of Environment	IG, IS
1	192	11	1.35	.56	.01	.13	Mixed organizations led by Ministry of Health and Welfare	IG, IS
2	165	22	2.08	.67	.02	.69	Mixed organizations led by CMMH and KCDC	IG, IS
3	111	6	1.24	.69	.01	.00	Mixed organizations in Gyeonggi Province	IG, IS
4	95	23	2.50	.80	.03	.22	Mixed organizations (Ministry of Public Safety & Security)	IG, IS
5	92	14	1.72	.65	.02	.00	Mixed organizations in Seoul Metropolitan City	IG, IS
6	74	5	1.21	.75	.02	.02	Mixed organizations in North Gyeongsang Province	IG
7	52	4	1.17	.85	.02	.00	Mixed organizations in Gangwon Province	IG
8	48	4	1.18	.85	.07	.15	Mixed organizations in Busan Metropolitan City	IG
9	47	7	1.41	.73	.09	.18	Mixed organizations in North Jeolla Province	IG
10	33	9	1.23	.56	.05	.14	Mixed organizations in Pyeongtaek city in Gyeonggi	IG, IS
11	29	19	2.69	.91	.20	.50	Mixed organizations (health-focused)	IS
12	27	7	1.10	.57	.45	.77	Mixed organizations (supportive organizations)	IS
13	24	2	0.17	.25	.05	.00	Local fire in Seoul Metropolitan City	IGF
14	19	2	0.21	.30	.05	.00	Local fire in South Gyeongsang Province	IGF
15	19	5	0.93	.58	.49	.80	Consulates, embassies (foreign affairs)	INTFA
16	18	2	0.21	.31	.06	.00	Local fire in North Gyeongsang Province	IGF
17	17	2	0.22	.32	.06	.00	Local fire in Gangwon Province	IGF
18	16	2	0.23	.34	.06	.00	Local fire in South Chungcheong Province	IGF
19	15	2	0.24	.35	.07	.00	Local fire in South Jeolla Province	IGF
20	12	2	0.29	.41	.08	.00	Local fire in Gyeonggi Province (North)	IGF
21	12	2	0.29	.41	.08	.00	Local fire in Busan Metropolitan City	IGF
22	12	2	0.29	.41	.08	.00	Local fire in North Chungcheong Province	IGF
23	11	2	0.30	.44	.09	.00	Local fire in North Jeolla Province	IGF
24	9	2	0.35	.50	.11	.00	Local fire in Incheon Metropolitan City	IGF
25	9	2	0.35	.50	.11	.00	Local fire in Daegu Metropolitan City	IGF
26	3	2	0.64	.92	.33	.00	Ministry of Unification (MOU)	INTFA

*Note*. High *H* and *E* values represent more diverse communities and high *D* and *C* values represent densely connected communities. IG = intergovernmental relations (between local, provincial, and national government relations); IS = intersectoral relations (between governmental, private, nonprofit, and civic organizations); IGF = intergovernmental relations among fire organizations; INTFA = international relations among foreign affairs organizations.

**Table 3.** Summary of Community Analysis (Qualitative Description).

Division	Community ID	Nature of Response Activities	Geographically Confined?	Functionally Similar?	Interorganizational Relations Appeared^a^	Diversity	Connectivity
Core response communities	1, 2	Direct	No	No	IG and IS	High	Low
Supportive functional communities	0, 4	Indirect	No	No	IG and IS	High	Low
	3, 5–10	Indirect	Yes	No	IG and IS	High	Low
	11, 12, 15, 26	Indirect	No	Yes	IS or INTFA	High	High
	13–14, 16–25	Indirect	Yes	Yes	IGF	Low	Low

*Note*. IG = intergovernmental relations; IS = intersectoral relations; INTFA = international relations (foreign affairs); IGF = intergovernmental relations (fire).

Figure 1 presents the MERS response network with communities in different colors to show the organizations’ clustering using ForceAtlas2 layout in Gephi. In Figure 2, the network’s community structure is clear to the human eye. From the figures (and the community analysis in Table 2), we find that the MERS response network was divided into two sets of communities according to which communities were at the center of the network and their nature of activity in the response, core response communities in one group and supportive functional communities in the other.

**Figure 3.**
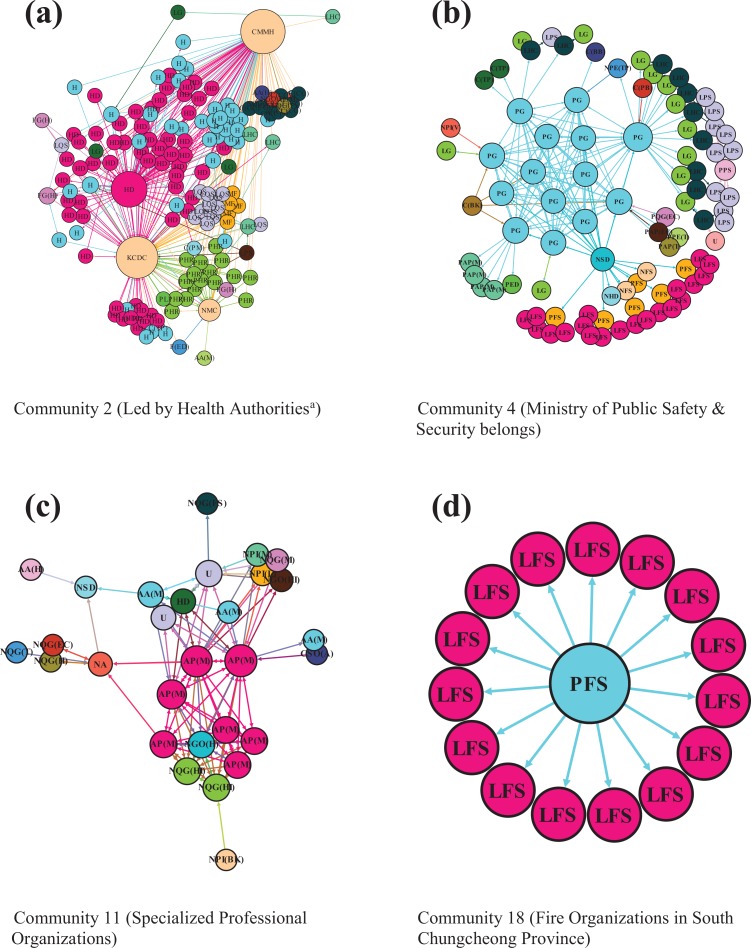
Inside selected communities. The three health organizations (Korea Centers for Disease Control and Prevention, Central MERS Management Headquarters, and the National Medical Center [NMC]) display organization names (not group IDs) for a presentation purpose.

The two core Communities (1 and 2) at the center of the response network included a large number of organizations, with a knot of intergroup coordination among the groups surrounding those two. These communities included organizations across government jurisdictions, sectors, and geographic locations (Table 2, description) and were actively involved in the response during the MERS outbreak. While not absolute, we observe that the network of a dominating organization had a “mushroom” shape of interactions with other organizations within the communities (also see Figure 3a). The dominant organizations were the central government authorities such as the MOHW, the CMMH, and KCDC. The national health authorities led the MERS response.

Other remaining communities were (1) confined geographically, (2) oriented functionally, or (3) both. First, some communities consisted of diverse organizations in the areas where two MERS hospitals are located—Seoul Metropolitan city and Gyeonggi Province (Communities 3 and 5). Organizations in these communities span government levels and sectors within the areas affected. Second, two communities consisted of organizations with different functions and performed supportive activities (Community 4, also see Figure 3b). Other supportive functional communities that focus on health (Community 11, see Figure 3c) or foreign affairs (Community 15) had a “spiderweb” shape of interactions among organizations within the communities. Third, several communities consisted of a relatively small number of organizations connected to one in the center (Communities 16, 17, 18, and 19). These consisted of local fire organizations in separate jurisdictions (see Figure 3d) that were both confined geographically and oriented functionally.

### Internal Structure and Interorganizational Relations Within the Communities

Table 2 summarizes the characteristics of the 27 communities in the response network. In Table 2, we also note distinct interorganizational relations present within the communities. The two core response communities include both intergovernmental and intersectoral relations.^7^ That is, organizations across government jurisdictions or sectors were actively involved in response to the epidemic in the communities. While diverse organizations participated in these core communities, the central government agencies led and directed other organizations, which reduced member connectivity.

Among the supportive functional communities, those that are confined geographically showed relatively high diversity but low connectivity (Communities 3, 5, and 6 through 10). These communities included intergovernmental relations within geographic locations. Secondly, communities of organizations with a specialized function showed relatively high diversity or connectivity. These included organizations from governmental and nongovernmental sectors and had no leading or dominating organizations. For example, Communities 11 and 12 had intersectoral relations but no intergovernmental relations. Thirdly, within each community of fire organizations in different geographic locations, one provincial or metropolitan fire headquarters was linked to multiple local fire stations in a star network. These communities, labeled IGF, had low member diversity and member connectivity, while they were organizationally and functionally coherent.

Table 3 summarizes the results elaborated above. In addition to the division of communities along the lines of the nature of their response activities, we observe that the structural characteristics of communities with only intersectional or international relations showed high diversity and high connectivity. Whenever intergovernmental relations were present in communities, however, the member connectivity was low, even if intersectoral relations appeared together within them.

## Discussion

We use the community detection method to gain a better understanding of the patterns of associations among diverse response organizations in an epidemic response network. The large data sets available and increased computational power significantly transform the study of social networks and can shed light on topics such as cohesive subgroups in large networks. Network studies today involve mining enormous digital data sets such as collective behavior online (Hagen et al., 2018), an e-mail corpus of a million messages (Tyler, Wilkinson, & Buberman, 2005), or scholars’ massive citation data (Kim & Zhang, 2016). The scale of ERNs in large disasters and emergencies is noteworthy (Moynihan, 2009; Waugh, 2003), and over 1,000 organizations appeared in Butts et al. (2012) study as well as in this research. Their connections reflect both existing structural forms by design and by emergent needs. The computational power needed to analyze such large relational data is ever higher and the methods simpler now, which allows us to learn about the entire network.

We find two important results. First, the national public health ERN in Korea split largely into two groups. The core response communities’ characteristics were that (1) they were not confined geographically, (2) organizations were heterogeneous across jurisdictional lines as well as sectors, and (3) the community’s internal structure was sparse even if intersectoral relations were present. On the other hand, supportive functional communities’ characteristics were that (1) they were communities of heterogeneous organizations in the areas affected that were confined geographically; (2) the communities of intersectoral, professional organizations were heterogeneous, densely connected, and not confined geographically; and (3) the communities of traditional emergency response organizations (e.g., fire) were confined geographically, homogeneous, and connected sparsely in a centralized fashion.

These findings show distinct features of the response to emerging infectious diseases. The core response communities suggest that diverse organizations across jurisdictions, sectors, and functions actually performed active and crucial MERS response activities. However, these organizations’ interaction and coordination inside the communities were found to be top down from the key national health authorities to all other organizations. This observation does not speak to the quality of interactions in the centralized top-down structure, but one can also ask how effective such a structure can be in a setting where diverse organizations must share authority, responsibilities, and resources. Second, infectious diseases spread rapidly and can break out in multiple locations simultaneously. The subgroup patterns in response networks to infectious diseases can differ from those of location-bound natural disasters such as hurricanes and earthquakes. While some organizations may not be actively or directly involved in the response, communities of these organizations can be formed to prepare for potential outbreaks or provide support to the core response communities during the event.

Second, we also find that the communities’ internal characteristics (diversity and connectivity) differed depending upon the types of interorganizational relations that appeared within the communities. Based on these analytical results, two propositions about the community structure in the ERN can be developed:

1. If intergovernmental relations operate in a community, the community’s member connectivity may be low, regardless of member diversity.
2. If community members are functionally similar,
  - professional organization communities’ (e.g., health or foreign affairs) member connectivity may be dense and
  - emergency response organization communities’ (e.g., fire) member connectivity may be sparse.

The results suggest that the presence of intergovernmental relations within the communities in ERNs may be associated with low member connectivity. However, this finding does not imply that those communities with intergovernmental relations are not organizationally or functionally cohesive. Instead, we may expect a different correlation between members’ functional similarity and their member connectivity depending upon the types of professions, as seen in 2(a) and (b).

Organizations’ concerted efforts during a response to an epidemic is a prevalent issue in many countries (Go & Park, 2018; Hodge, Gostin, & Vernick, 2007; Seo, Lee, Kim, & Lee, 2015; Swaan et al., 2018). The 2015 MERS outbreak in South Korea led to 16,693 suspected cases, 186 infected cases, and 38 deaths in the country (Korea Centers for Disease Control and Prevention, 2015). The South Korean government’s response to it was severely criticized for communication breakdowns, lack of leadership, and information secrecy (Korea Ministry of Health and Welfare, 2016).

The findings of this study offer a practical implication for public health emergency preparedness and response in the country studied. ERNs’ effective structure has been a fundamental question and a source of continued debate (Kapucu et al., 2010; Nowell, Steelman, Velez, & Yang, 2018). The answer remains unclear, but the recent opinion leans toward a less centralized and hierarchical structure, given the complexity of making decisions in disruptive situations (Brooks, Bodeau, & Fedorowicz, 2013; Comfort, 2007; Hart, Rosenthal, & Kouzmin, 1993). Our analysis shows clearly that the community structure and structures within communities in the network were highly centralized (several mushrooms) and led by central government organizations. Given that the response to the outbreak was severely criticized for its poor communication and lack of coordination, it might be beneficial to include more flexibility and openness in the response system in future events. We suggest taking advice from the literature above conservatively because of the contextual differences in the event and setting.

This study’s limitations also deserve mention. Several community detection methods have been developed with different assumptions for network partition. Some algorithms take deterministic group finding approaches that partition the network based on betweenness centrality edges (Girvan & Newman, 2002) or information centrality edges (Fortunato et al., 2004). Other algorithms take the optimization approaches we use in this article. In our side analyses, we tested three algorithms with the same data set: G-N, Louvain, and Leiden. The modularity scores were consistent, as reported in this article, but the number of communities in G-N and the other two algorithms differed. The deterministic group finding approach (G-N) found a substantively high number of communities. The modularity score can help make sense of the partition initially, but the approach is limited (Reichardt & Bornholdt, 2006). Thus, two questions remain: which algorithm do we choose and how do we know whether the community structure is robust (Karrer, Levina, & Newman, 2008)? In their nature, these questions do not differ from which statistical model to use given the assumptions and types of data in hand. The algorithms also require further examination and tests.

While we reviewed the data sources carefully multiple times to capture the response coordination, communication, and collaboration, the process of collecting and cleaning data can never be free from human error. It was a time-consuming, labor-intensive process that required trial and error. Further, the original written materials can have their own biases that reflect the source’s perspective. Government documents may provide richer information about the government’s actions but less so about other social endeavors. Media data, such as newspapers, also have their limitations as information sources to capture rich social networks. Accordingly, our results must be interpreted in the context of these limitations.

In conclusion, this article examines the community structure in a large ERN, which is a quite new, but potentially fruitful, approach to the field. We tested a rapidly developing analytical approach to the ERN to generate theoretical insights and find paths to exploit such insights for better public health emergency preparedness and response in the future. Much work remains to build and refine the theoretical propositions on crisis response networks drawn from this rich case study.

## Supplemental Material

Supplemental Material, Kim_Kim_Oh_et_al_Online_Supplement - Community Analysis of a Crisis Response Network Click here for additional data file. Supplemental Material, Kim_Kim_Oh_et_al_Online_Supplement for Community Analysis of a Crisis Response Network by Yushim Kim, Jihong Kim, Seong Soo Oh, Sang-Wook Kim, Minyoung Ku and Jaehyuk Cha in Social Science Computer Review

## Appendix

**Table A1.** Organizational Group ID and Description.

No.	Group ID	Description	Government	Sector
1	AA(H)	Academic associations (health)	Nongovernmental	Nonprofit
2	AA(M)	Academic associations (medical)	Nongovernmental	Nonprofit
3	AP(M)	Professional associations (medical)	Nongovernmental	Nonprofit
4	C(BB)	Hotels	Nongovernmental	Profit
5	C(BK)	Banks	Nongovernmental	Profit
6	C(CC)	Credit card companies	Nongovernmental	Profit
7	C(MT)	Telecommunication companies	Nongovernmental	Profit
8	C(MW)	Medical waste treatment companies	Nongovernmental	Profit
9	C(PB)	Public bath	Nongovernmental	Profit
10	C(PM)	Pharmaceutical industry	Nongovernmental	Profit
11	C(TP)	Transportation companies (Transportation)	Nongovernmental	Profit
12	CSO(A)	Civil society organizations (aging)	Nongovernmental	Nonprofit
13	CSO(H)	Civil society organizations (health)	Nongovernmental	Nonprofit
14	ES	Elementary schools	Nongovernmental	Public, profit
15	F(ED)	Foreign universities	N/A	N/A
16	FG(EC)	Foreign agencies (economics)	N/A	N/A
17	FG(FA)	Foreign embassies in Korea	N/A	N/A
18	FG(H)	Foreign agencies (health)	N/A	N/A
19	H	Hospitals	Nongovernmental	Profit
20	HD	Designated hospitals with isolated beds	Nongovernmental	Public, profit
21	INT(H)	International organizations	N/A	N/A
22	LA	Local assemblies	Governmental	Public
23	LAP(M)	Local professional associations (medical)	Nongovernmental	Nonprofit
24	LED	Local education offices	Governmental	Public
25	LFS	Local fire stations	Governmental	Public
26	LG	Local governments	Governmental	Public
27	LHC	Local health clinics	Governmental	Public
28	LPS	Local police stations	Governmental	Public
29	LQS	Local quarantine stations	Governmental	Public
30	MF	Clinical laboratories	Nongovernmental	Profit
31	NA	National assembly	Governmental	Public
32	NBH	The President	Governmental	Public
33	NFA	Korean embassies	Governmental	Public
34	NFS	National fire headquarters	Governmental	Public
35	NGO(F)	Nongovernment organizations (funeral)	Nongovernmental	Nonprofit
36	NGO(H)	Nongovernment organizations (health)	Nongovernmental	Nonprofit
37	NGO(HI)	Nongovernment organizations (claim adjuster)	Nongovernmental	Nonprofit
38	NHD	Central government health departments	Governmental	Public
39	NML	Military	Governmental	Public
40	NPE(TP)	Public enterprises (transportation)	Governmental	Public
41	NPI(BK)	Other public organizations (bank)	Governmental	Public
42	NPI(L)	Other public organizations (legal service)	Governmental	Public
43	NPI(M)	Other public organizations (mediation and arbitration)	Governmental	Public
44	NPI(V)	Other public organizations (Red Cross)	Governmental	Public
45	NPP	Political parties	Nongovernmental	Public
46	NPS	National Police Agency	Governmental	Public
47	NQG(EC)	Quasi-Governmental (economics)	Governmental	Public
48	NQG(F)	Quasi-Governmental (funeral culture and policy)	Governmental	Public
49	NQG(FR)	Quasi-Governmental (financial supervisory service)	Governmental	Public
50	NQG(FS)	Quasi-Governmental (fire)	Governmental	Public
51	NQG(H)	Quasi-Governmental (health)	Governmental	Public
52	NQG(HI)	Quasi-Governmental (health insurance)	Governmental	Public
53	NQG(M)	Quasi-Governmental (health-care accreditation)	Governmental	Public
54	NQG(T)	Quasi-Governmental (tourism)	Governmental	Public
55	NSD	Central government departments except health	Governmental	Public
56	PA	Provincial assemblies	Governmental	Public
57	PAP(EC)	Provincial professional association (economics)	Nongovernmental	Nonprofit
58	PAP(M)	Provincial professional association (medical)	Nongovernmental	Nonprofit
59	PAP(T)	Provincial professional association (tourism)	Nongovernmental	Nonprofit
60	PED	Provincial education offices	Governmental	Public
61	PFS	Provincial fire headquarters	Governmental	Public
62	PG	Provincial governments	Governmental	Public
63	PHR	Provincial health and environment research institutes	Governmental	Public
64	PL	Provincial prosecutors’ offices	Governmental	Public
65	PMHC	Provincial mental health centers	Governmental	Public
66	PPE(T)	Provincial public enterprises (tourism)	Governmental	Public
67	PPS	Provincial police agencies	Governmental	Public
68	PQG(EC)	Provincial Quasi-Governmental (economics)	Governmental	Public
69	U	Universities	Nongovernmental	Public, profit

## Notes

1. Sparse networks have fewer edges than the possible maximum number of edges within a network: *K* << *N* (*N −* 1)/2, where *K* is edges and *N* is nodes (Latora & Marchiori, 2001). The Katrina response network consisted of 1,577 organizations and 857 connections with a mean degree of approximately 1.1 (density 0.001).

2. Except for the quote, Comfort and Haase (2006) do not provide further explanation.

3. Incident Command System was established originally for the response to fire and has been expanded to other disaster areas (Moynihan, 2009; Waugh, 2003).

4.
*Kyunghyang Shinmun*, *Hankyoreh*, *Dong-a Ilbo*, and *Hankook Ilbo*.

5. In the second trial, we asked two graduate assistants to recollect the data separately from all news articles using the guideline based on our experience in the test round. In the end, we found that the process was not helpful because of the volume and redundancy of content in news articles different newspapers published, which is not an issue in analysis because it can be filtered and handled easily using network analysis tool. Because we had not confronted previous disaster response studies that collected network data from text materials, such as news articles and situation reports, and reported their reliability, we ended by developing the guideline and using it to construct the final data set from news articles.

6. We also classified organizations based on specialty, such as quarantine, economy, police, tourism, and so on regardless of jurisdictions. Twenty-seven specialty areas were classified. We note that the result of diversity analysis using the 27 specialty areas did not differ from that using the 69 organizational groups. The correlation of the diversity indices based on the two different classification criteria was *r* = .967. We report the result based on organization groups because the classification criterion can indicate better the different types of interorganizational relations than specialty in this context.

7. We did not measure the frequency, intensity, or quality of interorganizational relations but only the presence of either or both relations within the communities.
