# Supplementary material for: Community Analysis of a Crisis Response Network
Source: Soc Sci Comput Rev. 2019 Jul 28:0894439319858679. doi: 10.1177/0894439319858679 (PMC7206567; doi:10.1177/0894439319858679)
Supplement: Supplemental Material, Kim_Kim_Oh_et_al_Online_Supplement - Community Analysis of a Crisis Response Network [file Kim_Kim_Oh_et_al_Online_Supplement.pdf]

Supplemental Materials

Appendix A. Organizational Groups and Descriptions
Appendix B. Group IDs and the Number of Organizations in 27 Communities
Appendix C. Inside Selected Communities

Appendix A. Organizational Groups and Descriptions

No.	ID	Description	Gov.	Sector	Specialty	Org.(#)
1	AA(H)	Academic Associations (Health)	N	3	Health	2
2	AA(M)	Academic Associations (Medical)	N	3	Medical	10
3	AP(M)	Professional Associations (Medical)	N	3	Medical	9
4	C(BB)	Hotels	N	2	Hotel	1
5	C(BK)	Banks	N	2	Bank	2
6	C(CC)	Credit Card Companies	N	2	Credit Card	9
7	C(MT)	Telecommunication Companies	N	2	Telecommunication	3
8	C(MW)	Medical Waste Treatment Companies	N	2	Medical Waste	16
9	C(PB)	Public Bath	N	2	Bath	1
10	C(PM)	Pharmaceutical industry	N	2	Pharmaceuticals	2
11	C(TP)	Transportation Companies (Transportation)	N	2	Transportation	3
12	CSO(A)	Civil Society Organizations (Aging)	N	3	Population	1
13	CSO(H)	Civil Society Organizations (Health)	N	3	Health	1
14	ES	Elementary Schools	N	1,2	Education	1
15	F(ED)	Foreign Universities	N/A	N/A	Education	1
16	FG(EC)	Foreign Agencies (Economics)	N/A	N/A	Economy	2
17	FG(FA)	Foreign Embassies in Korea	N/A	N/A	Foreign Affair	1
18	FG(H)	Foreign Agencies (Health)	N/A	N/A	Health	17
19	H	Hospitals	N	2	Medical	119
20	HD	Designated Hospitals with Isolated Beds	N	1,2	Medical	63
21	INT(H)	International Organizations	N/A	N/A	Health	1
22	LA	Local Assemblies	G	1	Rule-making	1
23	LAP(M)	Local Professional Associations (Medical)	N	3	Medical	2
24	LED	Local Education Offices	G	1	Education	2
25	LFS	Local Fire Stations	G	1	Fire	205
26	LG	Local Governments	G	1	Government	227
27	LHC	Local Health Clinics	G	1	Health	255
28	LPS	Local Police Stations	G	1	Police	252
29	LQS	Local Quarantine Stations	G	1	Quarantine	13
30	MF	Clinical Laboratories	N	2	Medical	5
31	NA	National Assembly	G	1	Rule-making	1
32	NBH	The President	G	1	Government	1
33	NFA	Korean Embassies	G	1	Foreign Affair	2
34	NFS	National Fire Headquarters	G	1	Fire	2
35	NGO(F)	Non-Government Organizations (Funeral)	N	3	Funeral	1
36	NGO(H)	Non-Government Organizations (Health)	N	3	Health	1
37	NGO(HI)	Non-Government Organizations (Claim Adjuster)	N	3	Health Insurance	1
38	NHD	Central Government Health Departments	G	1	Health	5
39	NML	Military	G	1	Military	1
40	NPE(TP)	Public Enterprises (Transportation)	G	1	Transportation	2
41	NPI(BK)	Other Public Organizations (Bank)	G	1	Bank	1
42	NPI(L)	Other Public Organizations (Legal Service)	G	1	Law	1
43	NPI(M)	Other Public Organizations (Mediation and Arbitration)	G	1	Law	1
44	NPI(V)	Other Public Organizations (Red Cross)	G	1	Disaster	1
45	NPP	Political Parties	N	3	Politics	2
46	NPS	National Police Agency	G	1	Police	1
47	NQG(EC)	Quasi-Governmental (Economics)	G	1	Economy	1
48	NQG(F)	Quasi-Governmental (Funeral Culture and Policy)	G	1	Funeral	1
49	NQG(FR)	Quasi-Governmental (Financial Supervisory Service)	G	1	Finance	1
50	NQG(FS)	Quasi-Governmental (Fire)	G	1	Fire	1
51	NQG(H)	Quasi-Governmental (Health)	G	1	Health	3
52	NQG(HI)	Quasi-Governmental (Health Insurance)	G	1	Health Insurance	2
53	NQG(M)	Quasi-Governmental (Healthcare Accreditation)	G	1	Medical	1
54	NQG(T)	Quasi-Governmental (Tourism)	G	1	Tourism	1
55	NSD	Central Government Departments Except Health	G	1	Government	26
56	PA	Provincial Assemblies	G	1	Rule-making	1
57	PAP(EC)	Provincial Professional Assoc. (Economics)	N	3	Economy	1
58	PAP(M)	Provincial Professional Assoc. (Medical)	N	3	Medical	4
59	PAP(T)	Provincial Professional Assoc. (Tourism)	N	3	Tourism	1
60	PED	Provincial Education Offices	G	1	Education	3
61	PFS	Provincial Fire Headquarters	G	1	Fire	19
62	PG	Provincial Governments	G	1	Government	17
63	PHR	Provincial Health and Environment Research Institutes	G	1	Health	17
64	PL	Provincial Prosecutors' Offices	G	1	Law	2
65	PMHC	Provincial Mental Health Centers	G	1	Medical	14
66	PPE(T)	Provincial Public Enterprises (Tourism)	G	1	Tourism	1
67	PPS	Provincial Police Agencies	G	1	Police	16

68	PQG(EC)	Provincial Quasi-Governmental (Economics)	G	1	Economy	1
69	U	Universities	N	1,2	Education	10

Note: Gov. Column: Governmental (G), Non-Governmental (NG), Not Applicable (N/A); Sector Column: Public (1), Profit (2), Non-profit (3), Not Applicable (N/A)

Appendix B. Group IDs and the Number of Organizations in 27 Communities

ID	Group ID: # of Orgs
0	C(MW): 16, LPS: 98, NSD: 1, PPS: 9, LHC: 103, H: 1, LG: 6
1	LG: 85, NQG(H): 2, H: 73, U: 5, NPP: 2, PMHC: 14, AA(M): 5, NSD: 2, NHD: 1, AP(M): 1, HD: 2
2	NQG(F): 1, U: 1, FG(H): 3, NQG(FR): 1, LHC: 4, AA(H): 1, F(ED): 1, LQS: 13, AP(M): 1, PHR: 16, LPS: 1, LG: 3, C(TP): 1, PL: 1, C(CC): 9, C(PM): 1, AA(M): 1, H: 39, NHD: 3, NGO(F): 1, MF: 5, HD: 58
3	PHR: 1, LPS: 40, PPS: 1, LHC: 40, H: 2, LG: 27
4	NPE(TP): 1, PAP(M): 4, C(BK): 1, PED: 1, U: 1, PPS: 1, LHC: 10, C(BB): 1, C(PB): 1, LFS: 20, NPI(V): 1, LPS: 11, PAP(EC): 1, PFS: 6, NHD: 1, LG: 12, PQG(EC): 1, C(TP): 2, PG: 14, PPE(T): 1, NSD: 1, PAP(T): 1, NFS: 2
5	PA: 1, LPS: 30, PG: 1, H: 4, ES: 1, LG: 23, PED: 1, LED: 1, NML: 1, C(PM): 1, NSD: 1, PPS: 1, LHC: 24, HD: 2
6	LHC: 25, PG: 1, LG: 23, LPS: 24, PPS: 1
7	LHC: 17, LG: 17, LPS: 17, PPS: 1
8	LHC: 16, LG: 16, LPS: 15, PPS: 1
9	PG: 1, LPS: 15, PPS: 1, LHC: 14, LG: 14, PED: 1, PL: 1
10	C(BK): 1, LPS: 1, LG: 1, LED: 1, PFS: 1, LFS: 23, LHC: 2, LAP(M): 2, LA: 1
11	NPI(L): 1, NGO(HI): 1, NPI(M): 1, NPI(BK): 1, NQG(EC): 1, U: 2, NQG(FS): 1, NQG(HI): 2, NGO(H): 1, AA(H): 1, AP(M): 7, NQG(H): 1, NQG(M): 1, NQG(T): 1, CSO(A): 1, AA(M): 3, NSD: 1, NA: 1, HD: 1
12	NPE(TP): 1, C(MT): 3, NSD: 19, CSO(H): 1, NPS: 1, U: 1, NBH: 1
13	PFS: 1, LFS: 23
14	PFS: 1, LFS: 18
15	NFA: 2, FG(FA): 1, INT(H): 1, AA(M): 1, FG(H): 14
16	PFS: 1, LFS: 17
17	PFS: 1, LFS: 16
18	PFS: 1, LFS: 15
19	PFS: 1, LFS: 14
20	PFS: 1, LFS: 11
21	PFS: 1, LFS: 11
22	PFS: 1, LFS: 11
23	PFS: 1, LFS: 10
24	PFS: 1, LFS: 8
25	PFS: 1, LFS: 8
26	NSD: 1, FG(EC): 2

Note: Government entities start with L (Local level, red font), P (Provincial/metropolitan level, green font), or N (National level, blue font) for the first character of the organization descriptors. Otherwise, the descriptor implies non-governmental organizations, such as private companies (C), academic associations (AA), professional associations (AP), or organizations outside the country (INT or FC(H)). Some actors' service is presented within parenthesis, such as C(TP) for transportation companies.

Appendix C. Inside Selected Communities

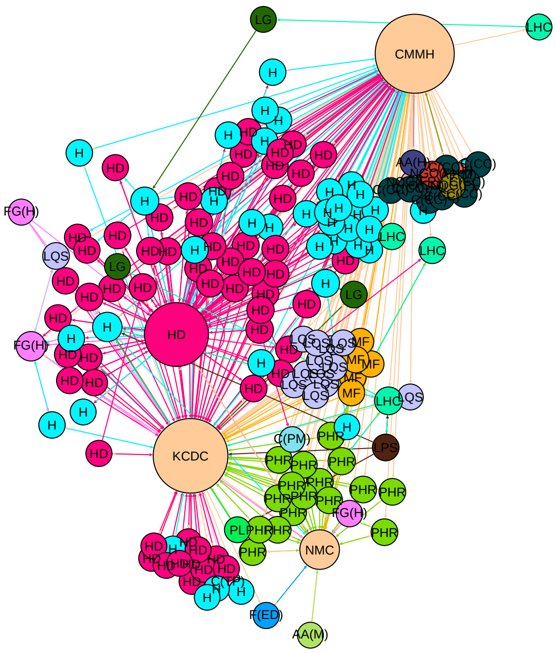

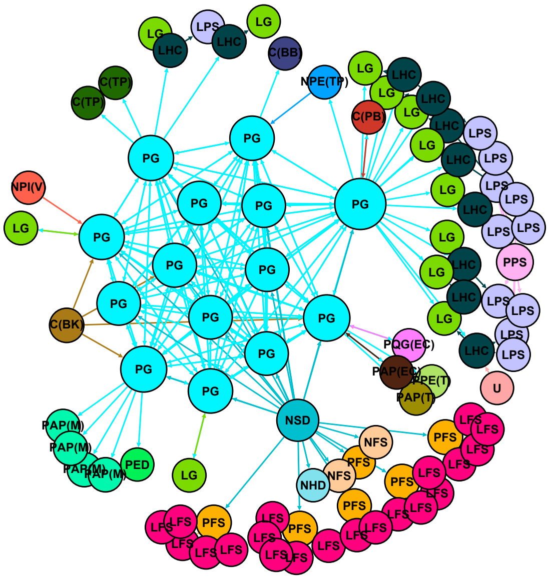

D1: Community 2 (Led by Health Authorities)

D2: Community 4 (Ministry of Public Safety & Security)

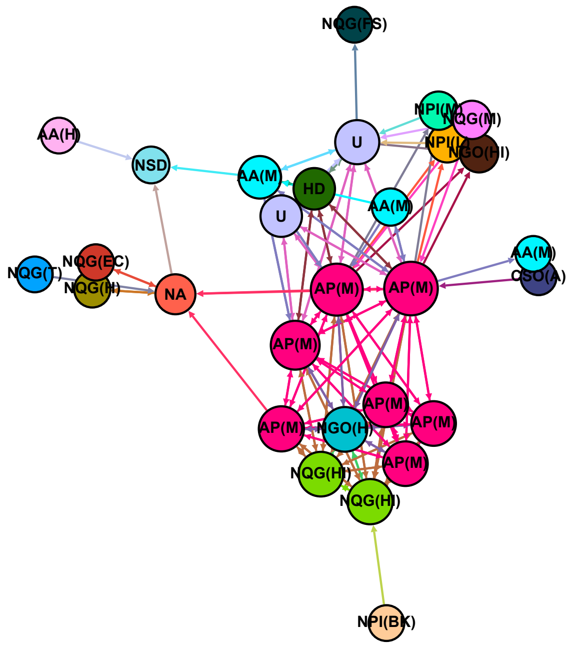

D3: Community 11 (Specialized Professional Organizations)

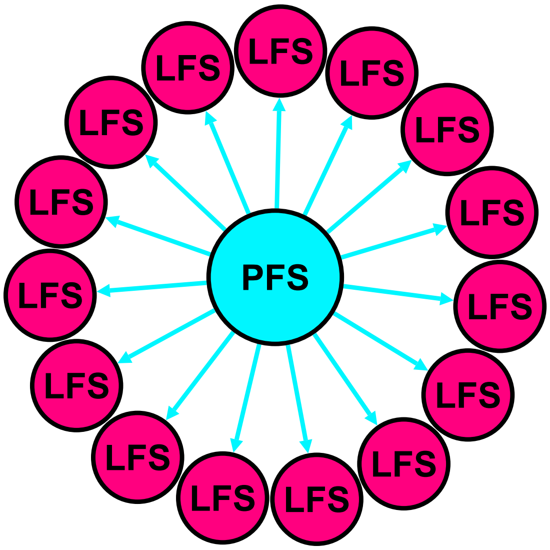

D4: Community 18 (Fire Organizations in South Chungcheong Province)
